# Arbuscular Mycorrhizal Fungi Orchestrate Soil Microbial Community Assembly Along a *Salix cupularis* Restoration Chronosequence in a Desertified Alpine Grassland

**DOI:** 10.1002/ece3.73133

**Published:** 2026-02-25

**Authors:** Xueqi Cai, Xingpeng Hu, Fei Yan, Dongming Chen, Bingxue Xiao, Xin Zheng, Kangqi Zhang, Jiqiong Zhou, Zhouwen Ma, Feida Sun, Yan Peng, Xiao Ma, Jeyakumar Paramsothy, Ran Xue, Lin Liu

**Affiliations:** ^1^ College of Grassland Science and Technology Sichuan Agricultural University Chengdu Sichuan People's Republic of China; ^2^ Sichuan Academy of Grassland Sciences Aba Sichuan People's Republic of China; ^3^ Environmental Sciences Group, School of Agriculture and Environment Massey University Palmerston North New Zealand

**Keywords:** AMF, co‐occurrence networks, desertified alpine meadow, microbial community assembly, vegetation restoration

## Abstract

Belowground microbes are emerging targets for ecosystem restoration. Understanding the assembly mechanisms of these microbial communities is critical for predicting ecosystem trajectories and optimizing restoration interventions. Arbuscular mycorrhizal fungi (AMF) are hypothesized to be key drivers of these eco‐evolutionary dynamics as a crucial and unique functional group associating with approximately 80% of terrestrial plant species. However, relatively little empirical information is available on the role of AMF in the soil microbial community assembly. Here, we used *Salix cupularis*, a native pioneer shrub species of desertified alpine meadows, to investigate the temporal dynamics of soil rhizosphere microbial communities across a restoration chronosequence (5, 10, and 20 years), with a particular focus on the AMF community. The results showed that minimal changes occurred in bacterial community structure, whereas fungal community exhibited more pronounced shifts along the chronosequence. Bacterial community assembly was initially deterministic and then became stochastic, while fungal assembly was consistently stochastic. Shrub planting enhanced the complexity of both bacterial and fungal networks over time. Co‐occurrence networks and Pearson correlation analysis revealed the “time‐dependent” regulatory role of the AMF community in soil microbial assembly. AMF acted as an orchestrator in the 10th year after planting (the edge density of AMF peaking at 15.0) prior to the transition to a stable, ECM‐dominated state in response to shifts in soil nutrient availability, particularly significant increases in MAOC and AP, as well as a decrease in DON. Our findings indicate that fungal communities exhibit higher sensitivity and highlight the dynamic regulatory function of AMF, especially under dual‐mycorrhizal symbiosis. These results provide novel mechanistic insights into soil microbe trajectories, suggesting that targeted AMF inoculation is crucial for the early‐to‐mid establishment phase of restoring desertified alpine meadows.

## Graphical abstract

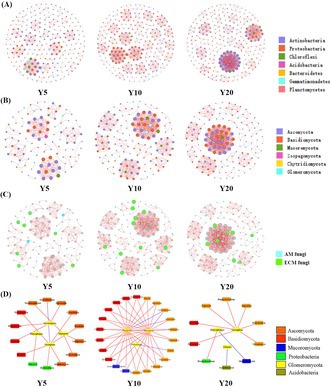

## 1 Introduction

As a global climate regulator (Wei et al. 2022) and a biodiversity hotspot (Li et al. 2020), the Qinghai‐Tibetan Plateau has experienced severe grassland degradation and desertification due to anthropogenic climate change (Zhou et al. 2024; Du et al. 2025) in recent decades, underscoring the urgency of restoring ecosystems (Guo et al. 2019). Human interventions such as vegetation restoration are a standard approach for rapidly enhancing ecological functions (Zhang et al. 2022). Since 1991, the Chinese government has proposed the National Plan on Combating Desertification (Wang et al. 2012). *Salix cupularis, a native shrub,* has been widely utilized as a pioneer plant due to its superior ecological adaptability and lower planting costs compared to grasses (Li, Shen, et al. 2021; Li et al. 2025). Many studies reported the role in regulating soil nutrients (Hu et al. 2018; Yan et al. 2025), moisture conservation (Hu et al. 2018; Li, Shen, et al. 2021), and understory vegetation (Li et al. 2022). However, ecosystem restoration efforts have so far been largely limited to monitoring aboveground plant communities. Belowground microbes with functional traits that enhance the resilience and complexity of ecosystems are emerging as targets for ecosystem restoration (Robinson et al. 2023) and warrant increasing attention. Therefore, the limited understanding about the contribution of pioneer shrubs to microbes and microbe–microbe interactions hampers progress in upscaling restoration.

Soil microbial communities serve as sensitive early indicators of soil degradation or amelioration (Hu et al. 2020). Shrubs reshape soil microbial communities through multiple pathways, including nutrient acquisition (root exudates, plant litter) (Liu et al. 2023), resource competition (water, light, soil nutrients) (Wang et al. 2019; Hu et al. 2021), and microenvironment regulation (canopy shading, windbreak and sand fixation) (Aanderud et al. 2008; Dong et al. 2023). However, restoring a microbial community is not simply a recombination of species, but a dynamic reconstruction of assembly processes and interspecific interactions. Theoretical and empirical studies consistently indicate that both stochastic processes and deterministic processes are the main factors driving soil microbial communities (Zhang et al. 2016; Chen et al. 2025). Although separate studies have detected the assembly or networks of microbial communities in different dominant plants (Wang, Zhu, et al. 2024; Yang, Wang, et al. 2024) and habitat conditions (Yao et al. 2018; Xu et al. 2021; Wang, Munson, et al. 2024), a comprehensive understanding of the ecological processes and their relative importance in the restoration chronosequence remains weak, particularly for key functional groups such as bacteria, fungi and arbuscular mycorrhizal fungi (AMF). Previous studies have reported that bacteria are more sensitive than fungi (Chen et al. 2022; Wang, Liu, et al. 2023; Shen et al. 2023). Fungi are generally considered to possess structural advantages, largely attributable to hyphal growth, osmotic regulation, and the protective functions of chitinous cell walls (Brown et al. 2020; Serafim et al. 2024; Napitupulu 2024). Whether microbial responses in semi‐arid ecosystems follow this pattern has not been systematically revealed yet.

As a special functional group, AMF associate obligately with approximately 80% of terrestrial plant species (Dong et al. 2021; Zhang et al. 2024), playing a crucial role in ecosystem functions and services (Gianinazzi et al. 2010; Solaiman et al. 2014). First, AMF contribute to plant growth in exchange for carbohydrates and lipids (Smith and Read 2008; Xu et al. 2024). Second, AMF mediate approximately 15% of soil total carbon to transfer into the ground in the QTP via their hyphae, which possess a faster rate than plant roots (Kaiser et al. 2015), and these carbons are essential for microbial metabolism (Wang et al. 2025). Additionally, AMF produce glomalin‐related soil protein (GRSP) to support the formation and stabilization of soil aggregates (Frey 2019), providing a suitable space for microbial survival. Furthermore, AMF convert photosynthetically derived carbons and release them as hyphal exudates, which alter the local pH value, phosphatase activity, and soil adhesiveness in the hyphosphere (Duan et al. 2024). Given that AMF are ubiquitous in grasslands (van der Heijden et al. 1998) and the keystone role identified in herbaceous vegetation restoration in the QTP heartland (Qin et al. 2019), we speculate that the dynamics of AMF are very likely to be the key pivot indicating and influencing the succession of the underground ecological interaction throughout the restoration chronosequence.

The existence of plants that form dual mycorrhizal associations is increasingly being recognized as a viable phenomenon in the natural environment at present (Smith and Read 2008; Teste et al. 2020; Rog et al. 2025), especially in Salicaceae (van der Heijden and Vosatka 1999; Baum et al. 2018). We suspect that *S. cupularis* might also establish such dual mycorrhizal symbiosis. In past studies, van der Heijden and Kuyper (2001) have discovered that even small amounts of AMF colonization were still beneficial to the shoot yield of *S. repens* despite more prolific ECM. Baum et al. (2018) reported that AMF have a strong *Salix* genotype dependence and functional enzymes‐regulating ability in an *Laccaria tortilis* (an ectomycorrhizal fungi)‐dominated environment. Therefore, it is generally believed that AMF might occupy a more critical ecological niche than ECM in the early restoration of nutrient‐poor and frequently disturbed systems (Teste et al. 2020; Xing 2024; Nash et al. 2025) due to its host‐generalist, low‐carbon cost and efficient acquisition for inorganic nutrients (Smith and Read 2008). However, it remains unclear how AMF community dynamics and their regulatory role in microbial interactions function unfold. Thus, increased explorations are needed, which contribute to a better understanding of restoration from a perspective of belowground ecosystems.

Here, we investigated soil microbial community dynamics in the rhizosphere of *S. cupularis* along a restoration chronosequence, with a particular focus on the role of AMF in microbial community assembly. Given the unique environmental constraints and the strong plant‐microbe coupling expected during shrub establishment, we proposed and tested the following hypotheses: (1) bacterial and fungal communities exhibit distinct sensitivities to restoration, and bacteria are more sensitive than fungi; and (2) *S. cupularis* exhibits dual‐mycorrhizal symbiosis with a time‐dependent shift of AMF role in microbial interactions.

## 2 Materials and Methods

### 2.1 Study Site

The experiment was carried out in the southeastern part of Qinghai‐Tibetan Plateau, 50 km west of Zoige County, Aba Tibetan and Qiang Autonomous Prefecture, Sichuan Province, China (33°43′N, 102°27′E), with an altitude of more than 3400 m. The climate of the study area is classified as a continental plateau cold humid monsoon type. The mean annual precipitation is 648.5 mm, concentrated mainly from May to October, and the potential evapotranspiration is about 782.8 mm. The mean annual temperature is 1.1°C; the average temperature in the coldest (January) and the hottest (July) months are −10.7°C and 10.7°C, respectively. The annual wind speed ranges from 1.7 to 3.2 m/s. Due to the wind erosion in this area, the soils are classified as arenosols (IUSS Working Group WRB 2022). Since 1998, cuttings of the native shrub *S. cupularis*, known for its strong tolerance and adaptability, have been successively planted in this area across different years. A plethora of 1 m × 1 m grids was woven by *S. cupularis* branches to establish a network‐like sand barriers. One current‐year *S. cupularis* shoot was planted as a cutting in the center of each grid.

### 2.2 Experimental Design and Sampling

**FIGURE A1:**
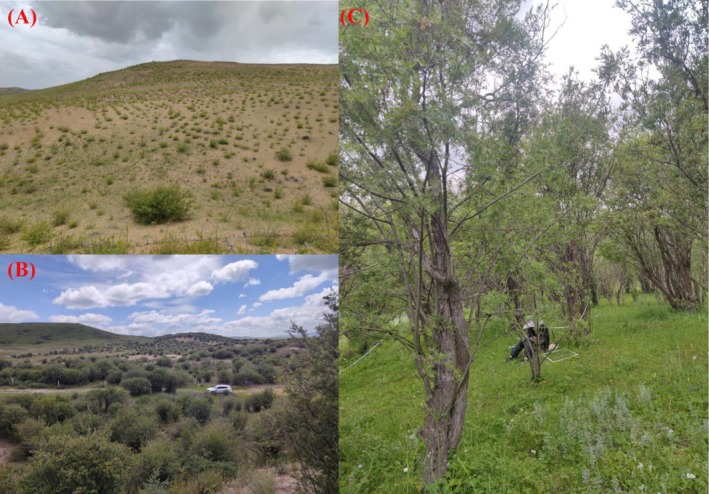
Vegetation landscape of *Salix cupularis* at three different planting years: (A) 5‐year‐old site showing sparse shrub coverage on sandy soil; (B) 10‐year‐old site with moderate woody plant density and increased ground cover; (C) 20‐year‐old site characterized by dense stands, developed canopy structure, and abundant understory vegetation. All photographs were taken by the authors during field sampling at 50 km west of Zoige County (33°43′N, 102°27′E).

This study employed a space‐for‐time substitution approach across a chronosequence defined by the planting age of *S. cupularis* (Figure A1). Since the experimental area was initially established using healthy one‐year‐old *S. cupularis* cuttings, stand age was calculated from the planting year (designated as Year 1). Consequently, we systematically selected three representative chronosequences to understand the restoration process through different restoration stages, including 5‐, 10‐, and 20‐planting year.

For each planting year, we established six independent plots, each measuring 40 m × 50 m (2000 m^2^). A minimum buffer distance of 200 m was maintained between plots to ensure spatial independence and reduce autocorrelation. All plots experienced comparable climatic conditions to minimize legacy effects. Rhizosphere soil sampling was conducted in July 2022 (vigorous growth period of *S. cupularis*). Within each plot, we randomly selected three healthy, standard‐sized *S. cupularis* individuals. Sampling tools (shovels, corers) were sterilized with 75% ethanol between samples, and fresh sterile gloves were used for each collection. We carefully excavated the root systems from a soil depth range of 20–40 cm, a critical zone characterized by active fine roots (Li et al. 2025). The excavated roots were gently shaken to remove loosely bound bulk soil. The rhizosphere soil, operationally defined as the soil tightly adhering to the root surface (within approximately 1–2 mm) (Phillips and Fahey 2007; Yang et al. 2023), was then collected from the absorptive fine roots (< 2 mm in diameter) using sterile soft‐bristle brushes. Soil samples from the three individuals within the same plot were pooled and thoroughly homogenized to form one composite sample per plot (*n* = 6 per restoration year). After manually removing visible plant residues, animal remains, litter, stones, and subsequently passing the soil through a 2‐mm sieve, the soil samples of each plot were evenly mixed and divided into three portions: frozen at −80°C for subsequent DNA extraction, at 4°C for microbial biomass determinations, and air‐dried for physicochemical characterization.

### 2.3 Soil Properties Analyses

Soil water content (SWC) was assessed by calculating the weight difference following 24 h of drying at 105°C ± 1°C. Soil pH was determined with a pH electrode in a suspension of soil and water. Soil organic carbon (SOC) was quantified through potassium dichromate (K_2_Cr_2_O_7_) oxidation. Both microbial biomass carbon (MBC) and microbial biomass N (MBN) were assessed through the chloroform fumigation‐potassium sulfate (K_2_SO_4_) extraction method (Brookes et al. 1985). The C obtained from non‐fumigated samples represented dissolved organic carbon (DOC). Dissolved organic N (DON) was calculated by subtracting total inorganic N from TDN. NH_4_^+^‐N and NO_3_^−^‐N were measured using colorimetric methods: NH_4_^+^‐N was measured as described by Sinsabaugh et al. (2000); while the NO_3_^−^‐N analysis protocol was adapted from the Nitrate Elimination Co. Inc. (NECi) Method N07‐0003 (http://www.nitrate.com/node/164). Available phosphorus (AP) was determined by the molybdenum blue colorimetric method after extraction by 0.5 M NaHCO_3_ digestion. Soils were separated by size into sand + particle associated carbon (POC) (> 53 μm) and silt + clay + mineral associated carbon (MAOC) (< 53 μm) following Cambardella and Elliott (1992). These fractions are hereafter referred to as “POC” and “MAOC,” respectively. Briefly, 5.75–6.25 g of 2‐mm sieved soil dried at 60°C was shaken for 18 h with 12 glass beads in 30 mL of 0.5% sodium hexametaphosphate to disrupt all aggregates. The resulting soil slurry was rinsed with DI water over a 53‐μm sieve to isolate POC and remove glass beads. Soil solution passing through the sieve was deemed MAOC. Both the POC and MAOC fractions were dried at 60°C until reaching constant mass. Recoveries of the initial soil masses in the summed fractions were between 96% and 105% for all samples, with a mean recovery of 101.7%. Soils and fractions containing carbonates (identified by effervescence after addition of 5% HCl, 36 samples) were treated to remove inorganic carbon via HCl fumigation (Harris et al. 2001).

### 2.4 DNA Extraction and Sequencing

Microbial genomic DNA was extracted from soil using the E.Z.N.A. soil DNA kit (Omega Bio‐tek, Norcross, GA, USA). The total DNA extracts were detected by 1% agarose gel electrophoresis. DNA was fragmented to approximately 400 bp using the Covaris M220, and PE libraries were constructed with the NEXTFLEX Rapid DNA‐Seq Kit. Finally. The processed DNA was then sent to the Illumina Novaseq platform (Meggie Company, Shanghai, China) for PE 150 sequencing.

### 2.5 Bioinformatics Analysis

Fastp v0.23.0 was used for quality filtering and optimization of the adapter sequences at the 3′ and 5′ ends of the reads. MEGAHIT v1.1.2 was utilized to splice and assemble the obtained clean reads. Prodigal v2.6.3 was used to predict the ORF of contig ≥ 300 bp in the splicing results. Genes with nucleic acid lengths ≥ 100 bp were selected and translated into amino acid sequences. CD‐HIT v4.6.1 was used to cluster gene sequence and build a non‐redundant gene set subsequently. The gene abundance was obtained using SOA Paligner soap2.21.

Using DIAMOND software to compare the amino acid sequences of non‐redundant gene sets with the non‐redundant protein library NR database (Comparison type: BLASTP). The taxonomic annotation was obtained through the taxonomic information database corresponding to the NR library. The sum of the gene abundances corresponding to each species was used to calculate species‐specific abundance. The abundance of species in each sample was counted at taxonomic levels, including domain, kingdom, phylum, class, order, family, genus, and species to construct an abundance spectrum at each corresponding taxonomic level.

### 2.6 Statistical Analysis

One‐way ANOVA was performed to detect the differences in soil physicochemical properties and microbial alpha diversity indices (bacteria, fungi, and AMF) at different restoration years, followed by Tukey's HSD or Tukey–Kramer post hoc tests (*p* < 0.05). The Tukey–Kramer test was specifically employed to account for unequal sample sizes, as the Pielou_e indices of two early‐stage (planting‐Y5) AMF samples were unavailable due to insufficient sequencing depth.

Based on Bray‐Curtis distance, Non‐metric Multidimensional Scaling (NMDS) was performed to visualize microbial beta diversity. Differences between groups were examined for statistical significance using permutational multivariate analysis of variance (PERMANOVA) via the adonis2 function. Furthermore, redundancy analysis (RDA) was conducted to identify the relationships between AMF community structure and soil physicochemical properties; variables were standardized during the analysis to eliminate the influence of different units.

Levins' niche width index (Levins 1968) was calculated using the “Spaa” package to evaluate the habitat specialization of soil bacteria and fungi (Yang, Zhao, et al. 2024). Additionally, to further quantify the relative importance of deterministic and stochastic processes in microbial community assembly, the NST value was calculated by the “NST” package. NST < 0.5 indicated dominance of deterministic processes, while NST > 0.5 suggested dominance of stochastic processes (Ning et al. 2019). Pearson correlation analysis was further executed to evaluate the relationships between AMF and other soil microbes. For all statistical analyses, *p* < 0.05 was considered statistically significant.

To determine fungal trophic mode categories, functional annotation was performed using the FUNGuild database, retaining only “probable” and “highly probable” confidence levels data. Based on random matrix theory (RMT), genera with a relative abundance > 0.025‰ were selected. Using the same threshold (Spearman correlation coefficient, *R* > 0.6, *p* < 0.05) to investigate the co‐occurrence pattern, microbial network data were generated by “Hmisc” and “psych” packages (Xiao et al. 2023), and visualized by Gephi 0.9.2 and Cytoscape 3.8.0 to identify microbial interactions. In this study, edge density was utilized as a proxy for network complexity (Banerjee et al. 2019; Wagg et al. 2019; Yuan et al. 2022).

All statistical analyses and visualizations were implemented in R (v.4.3.2) and Origin 2024.

## 3 Results

### 3.1 Soil Microbial Community Composition

**FIGURE 1:**
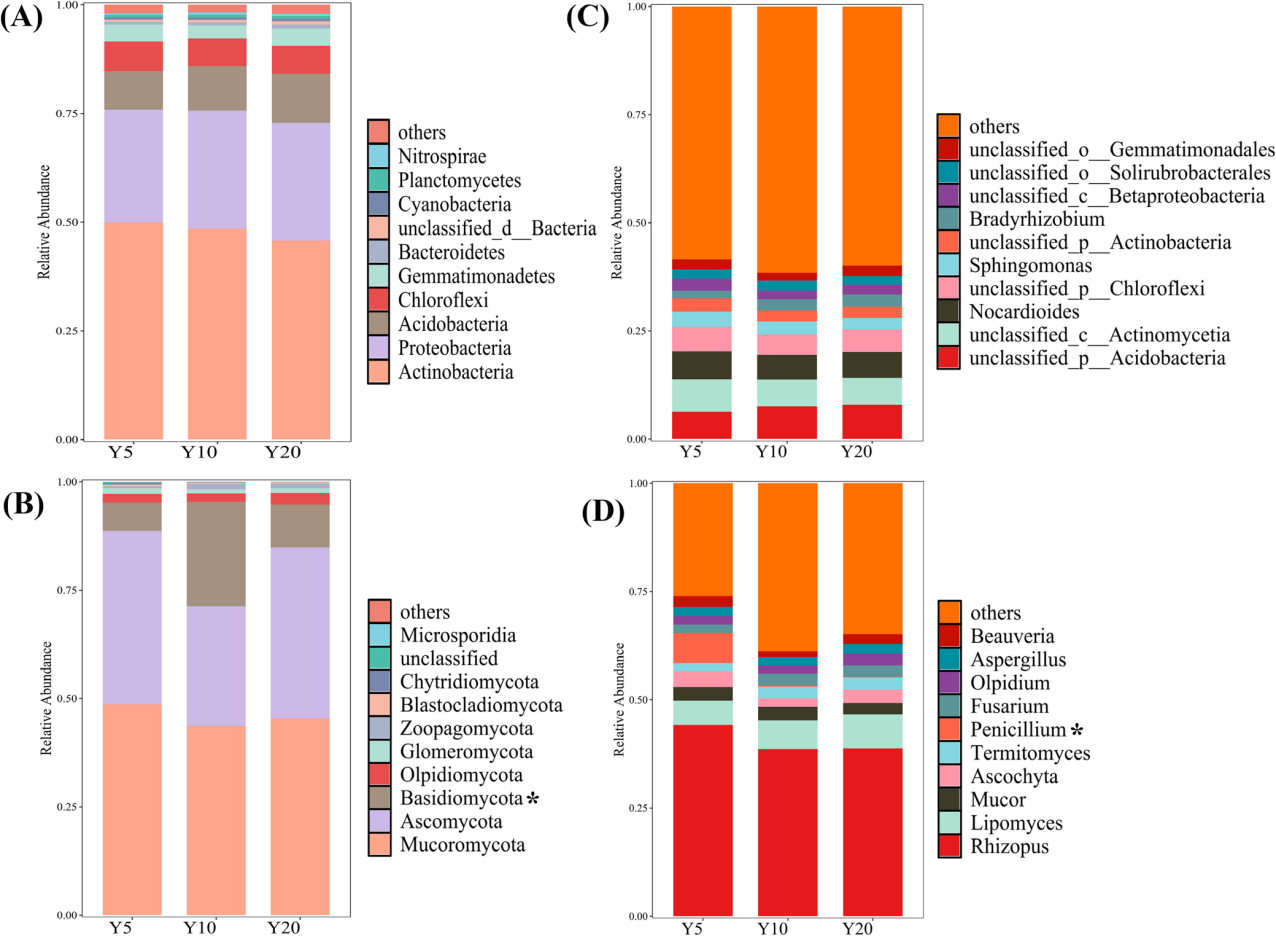
Relative abundances of dominant soil bacterial (A, C) and fungal (B, D) communities at the phyla and genus level along with restoration (Y5: Planting 5 years; Y10: Planting 10 years; Y20: Planting 20 years). Taxa with relative abundances in the top 10 are shown, the rest are merged into “other”. * indicates the significant difference at *p* < 0.05 level.

We identified 158 phyla, 268 classes, 474 orders, 978 families, 3857 genera, and 28,757 bacterial species, as well as 10 phyla, 34 classes, 87 orders, 208 families, 307 genera, and 529 fungal species in the soil samples. At the phylum level (Figure 1A,C), Actinobacteria (46%–50%), Proteobacteria (26%–27%), Acidobacteria (8%–11%) and Chloroflexi (6%–7%) dominated bacterial communities across the restoration chronosequence. Fungal communities were dominated by Mucoromycota (45%–49%) and Ascomycota (39%–40%). The relative abundance of Basidiomycota in the planting‐Y10 increased significantly to 24% (*p* < 0.05), replacing Ascomycota (12%) as a dominant phylum, while no significant differences were observed between planting‐Y5 and planting‐Y20. At the genus level (Figure 1B,D), dominant bacterial genera changed insignificantly, whereas the relative abundance of fungal genus *Penicillium* decreased by 20‐to 50‐fold in planting‐Y10 (*p* < 0.05), with no significant differences between planting‐Y5 and planting‐Y20.

**FIGURE 2:**
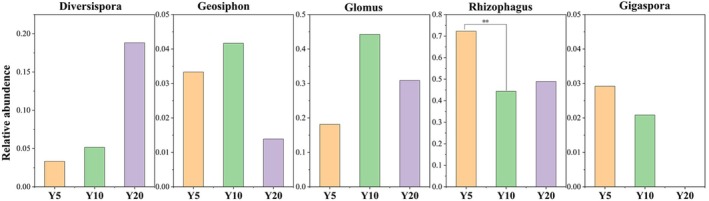
The composition of AMF community at the genus level along with restoration (Y5: Planting 5 years; Y10: Planting 10 years; Y20: Planting 20 years). ** indicates significant differences at *p* < 0.01 level. The absence of * indicates that there is no significant difference between the two sets of data in successive stages.

AMF (Glomeromycota) accounted for only 0.97%–1.3% of the fungal community, with only five genera detected across the chronosequence (Figure 2). Genus‐level composition varied substantially.** *Rhizophagus* remained dominant across all three restoration years, although its relative abundance decreased significantly in planting‐Y10. Conversely, *Glomus* peaked in planting‐Y10, increasing to 44% and emerging as a co‐dominant genus. *Diversispora* increased by 3.6‐to 6‐fold. *Geosiphon* exhibited a “hump‐shaped” pattern, whereas *Gigaspora* disappeared in planting‐Y20.

### 3.2 Soil Microbial Community Diversity

**FIGURE 3:**
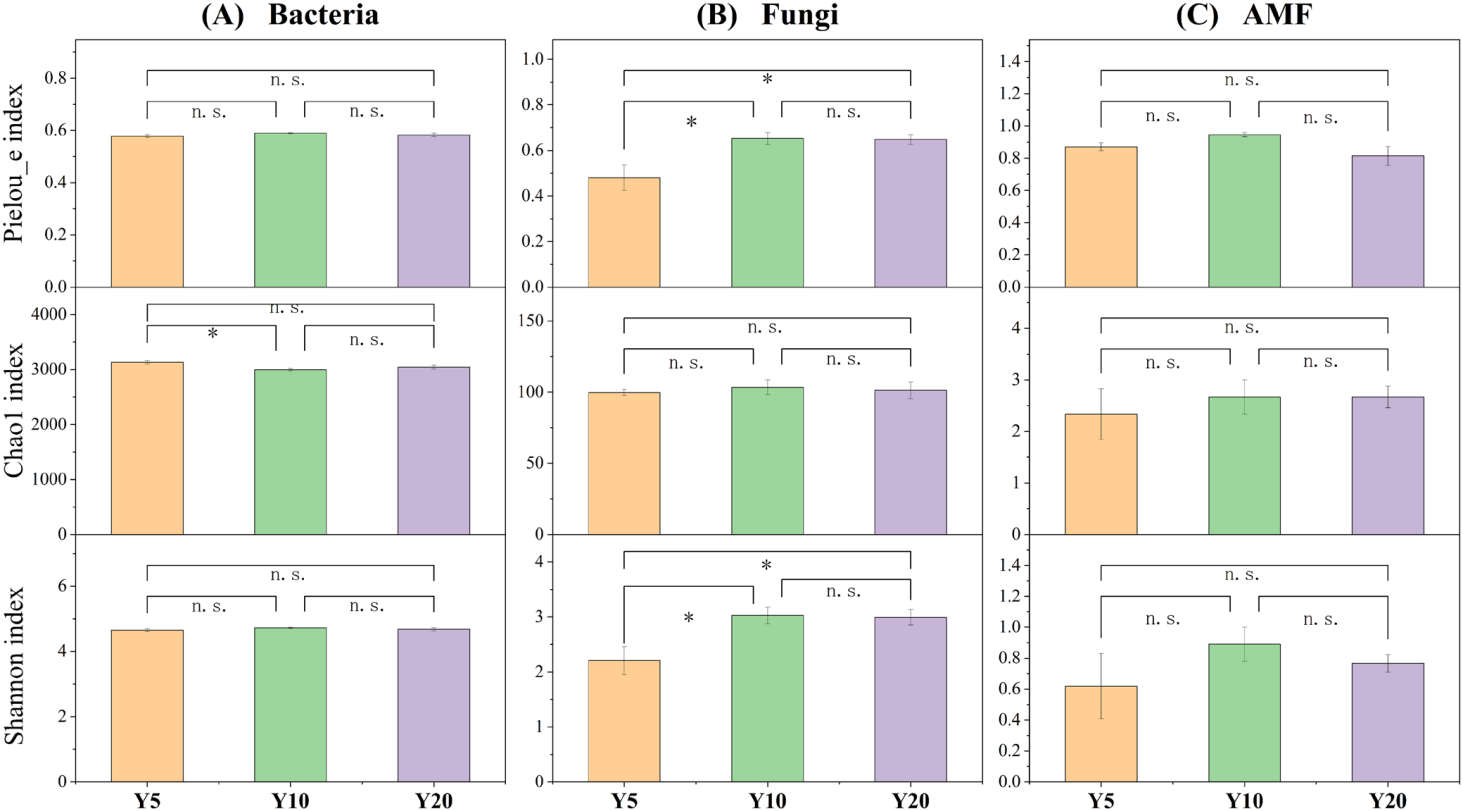
Alpha diversity of soil microorganisms along with restoration (Y5: Planting 5 years; Y10: Planting 10 years; Y20: Planting 20 years). (A) Bacteria, (B) Fungi, (C) AMF. Values are means ± SE (*n* = 6). * indicates the significant difference at *p* < 0.05 level, NS indicates no significant differences between successive stages.

**FIGURE 4:**
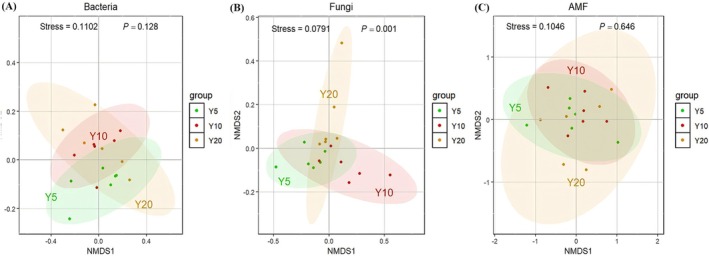
Beta diversity of soil microorganisms along with restoration (Y5: Planting 5 years; Y10: Planting 10 years; Y20: Planting 20 years). (A) Bacterica, (B) Fungi, (C) AMF. Stress < 0.2 indicates that NMDS has statistical significance, *p* < 0.05 indicates a significant difference. The shaded areas show the 95% confidence interval of the fit.

We quantified the richness and α‐diversity of soil bacteria, fungi, and AMF across the chronosequence (Figure 3). Bacterial richness was significantly higher in the planting‐Y5 (Chao1 index = 3133 ± 31) than in the planting‐Y10 (Chao1 index = 3000 ± 22) (*F*(2,15) = 5.06, *p* = 0.02) (Figure 3A). Fungal diversity indices were significantly higher in the planting‐Y10 (Shannon index = 2.20 ± 0.25; Pielou index = 0.48 ± 0.06) and planting‐Y20 (Shannon index = 3.01 ± 0.15; Pielou_e index = 0.65 ± 0.03) than in the planting‐Y5 (Shannon index = 2.99 ± 0.14; Pielou_e index = 0.64 ± 0.02) (*F*(2,15) = 6.07, *p* = 0.01; *F*(2,15) = 6.63, *p* = 0.008) (Figure 3B). AMF α‐diversity and richness showed a “hump‐shaped” variation trend with restoration (Figure 3C). NMDS revealed no significant differences in bacterial (*p* = 0.128) or AMF (*p* = 0.646) communities, whereas fungal communities were significantly separated (*p* = 0.001) (Figure 4). Fungal communities displayed high heterogeneity, while bacterial and AMF communities overlapped substantially, indicating relatively minor compositional shifts.

### 3.3 Soil Microbial Community Assembly

**FIGURE 5:**
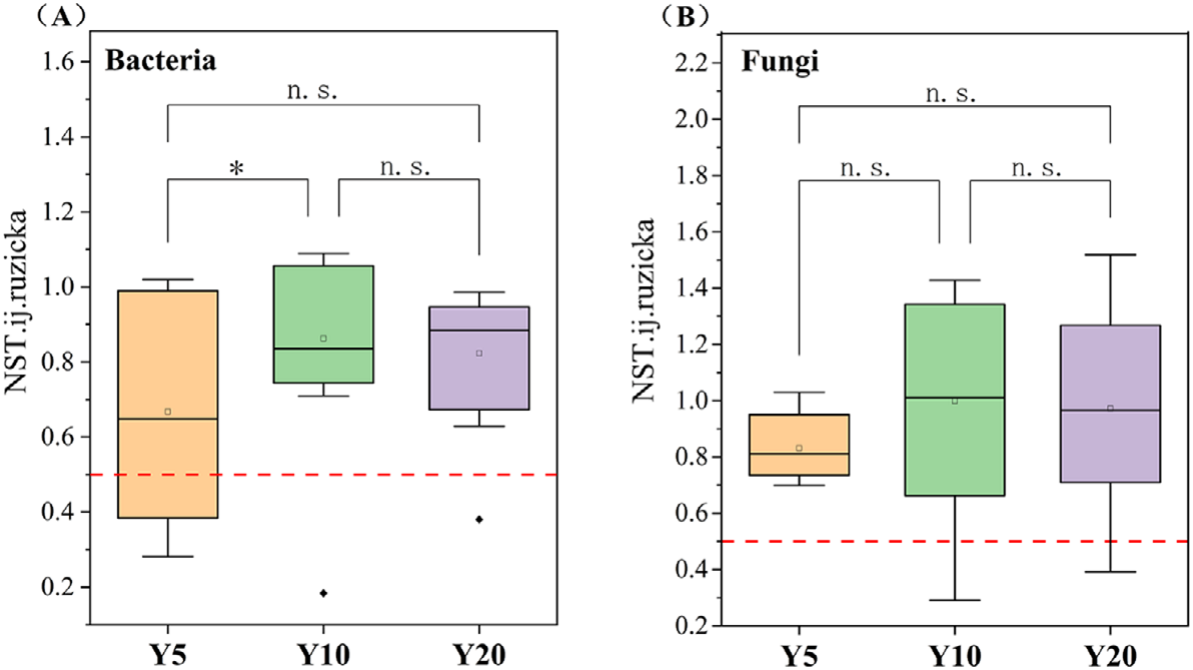
The microbial community assembly along with restoration (Y5: Planting 5 years; Y10: Planting 10 years; Y20: Planting 20 years). (A), the NST of bacteria; (B), the NST of fungi. Values are means ± SE (*n* = 6). * indicates the significant difference at *p* < 0.05 level. NST < 0.5 indicates dominance of deterministic processes, NST > 0.5 indicates dominance of stochastic processes.

Using normalized stochasticity ratio to quantify the relative contributions of stochastic and deterministic processes to community assembly, we detected that bacterial communities were driven by a combination of deterministic and stochastic processes in the planting‐Y5, but shifted towards predominantly stochastic assembly in the planting‐Y10 and planting‐Y20 restoration (Figure 5A), while fungal communities were dominated by stochastic processes consistently across the chronosequence (Figure 5B). Levins' niche width demonstrated that soil fungi had a narrower ecological niche compared to bacteria (*F* = 239.641, *p* < 0.001).

### 3.4 Soil Microbial Co‐Occurrence Network Analysis

**FIGURE 6:**
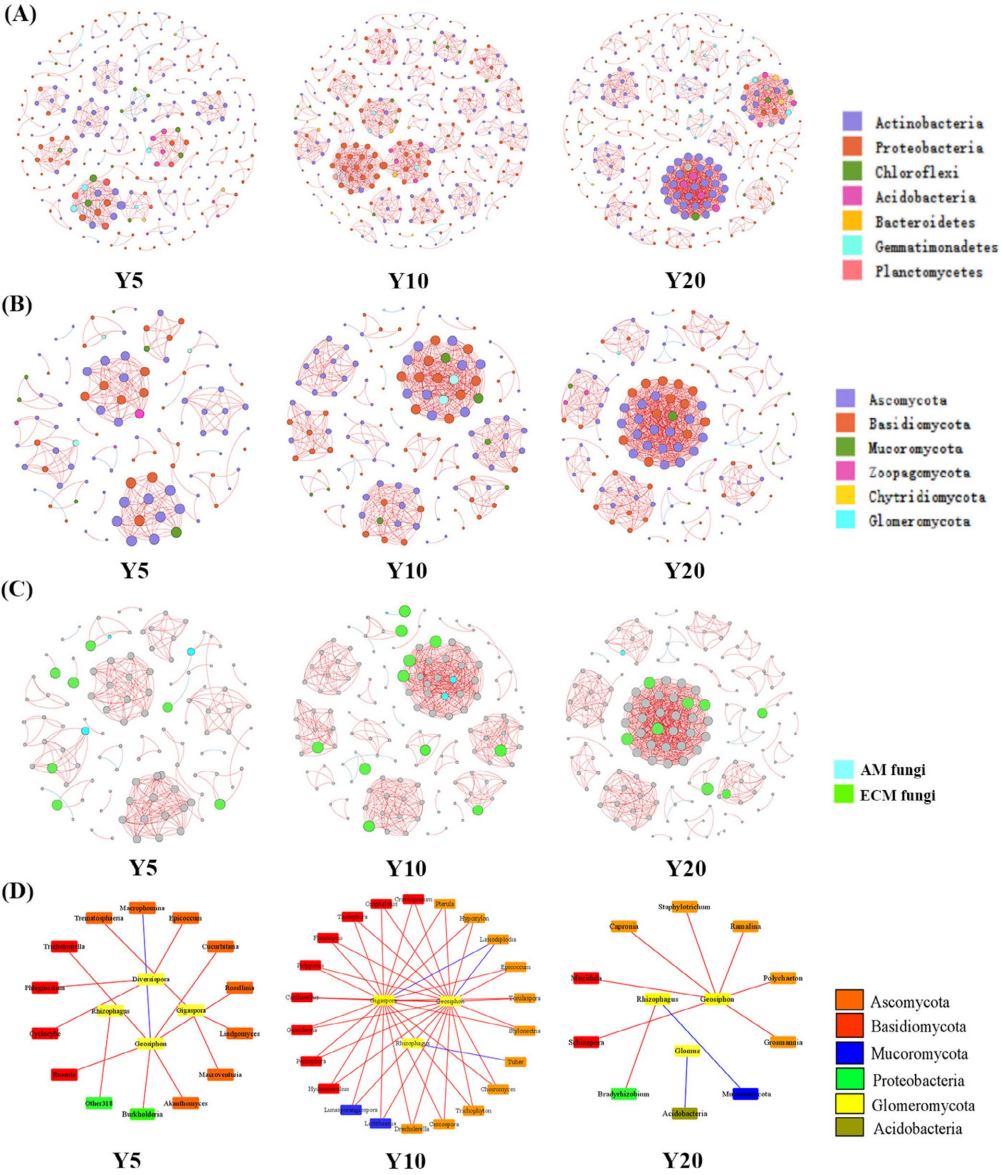
Co‐occurrence networks of soil microorganisms along with restoration (Y5: Planting 5 years; Y10: Planting 10 years; Y20: Planting 20 years). (A) bacterial networks; (B) fungal networks; (C) proportion of AMF and ECM fungi in fungal network. (D) networks built with AMF as the core. Each node is on behalf of a genus. All red lines indicate positive relationships between two individual nodes, and blue lines indicate negative.

Co‐occurrence networks were constructed to elucidate microbial interactions (Figure 6), with topological properties were detailed in Table 1. In the bacterial network, the number of links surged by 87% in the planting‐Y10 and 16% in the planting‐Y20. The average degree followed a similar trajectory, rising by 108% and 28% during these respective periods, whereas the average clustering coefficient changed slightly (0.3% and 1.2% increases) and modularity declined by 20% and 4% respectively. The fungal network exhibited a more rapid increase of links (overall 177%), but the average clustering coefficient fluctuated‐decreasing in the planting‐Y10 (by 2.5%) before recovering in the planting‐Y20 (2.2%), and modularity declined by 25% and 6.5% across the chronosequence. Positive correlations accounted for over 90% of total links in the networks. Bacterial networks consistently maintained higher connectivity and modularity than fungal counterparts throughout succession.

**TABLE 1:** Bacterial and fungal network properties at different stages of restoration.

Network parameters	Bacteria			Fungi		
	Y5	Y10	Y20	Y5	Y10	Y20
Number of nodes	255	292	285	110	145	154
Number of links	565	1059	1228	277	638	767
Average degree	3.233	6.709	8.618	5.036	8.80	9.961
Network diameter	4	6	2	2	4	2
Average density	0.014	0.025	0.03	0.046	0.061	0.065
Modularity	0.939	0.9	0.749	0.814	0.764	0.611
Average clustering coefficient	0.979	0.991	0.994	0.97	0.946	0.967
Average path length	1.218	1.764	1.006	1.028	1.023	1.015
Positive	92.2%	97.45%	98.29%	97.84%	98.9%	98.44%

Actinobacteria and Proteobacteria constituted the most abundant bacterial nodes (Figure 6A), while Ascomycota and Basidiomycota dominated the fungal networks (Figure 6B). Although low in relative abundance, AM and ECM fungi occupied distinct topological niches, manifesting in comparable edge densities in the planting‐Y5 (edge density = number of edges/number of nodes), with AMF edge density peaking at 15.0 in the planting‐Y10 before decreasing by approximately 4.5‐fold in the planting‐Y20. Conversely, ECM edge density steadily increased, reaching 20.38 by planting‐Y20 (Figure 6C). Interaction patterns further differentiated these groups: associations between AMF with major fungal phyla (Ascomycota, Basidiomycota, and Mucoromycota) strengthened initially but waned during restoration, whereas interactions with bacterial Proteobacteria and Acidobacteria remained consistently weak (Figure 6D).

### 3.5 The Relationships of AMF Diversity and Soil Microorganism Diversity

**FIGURE 7:**
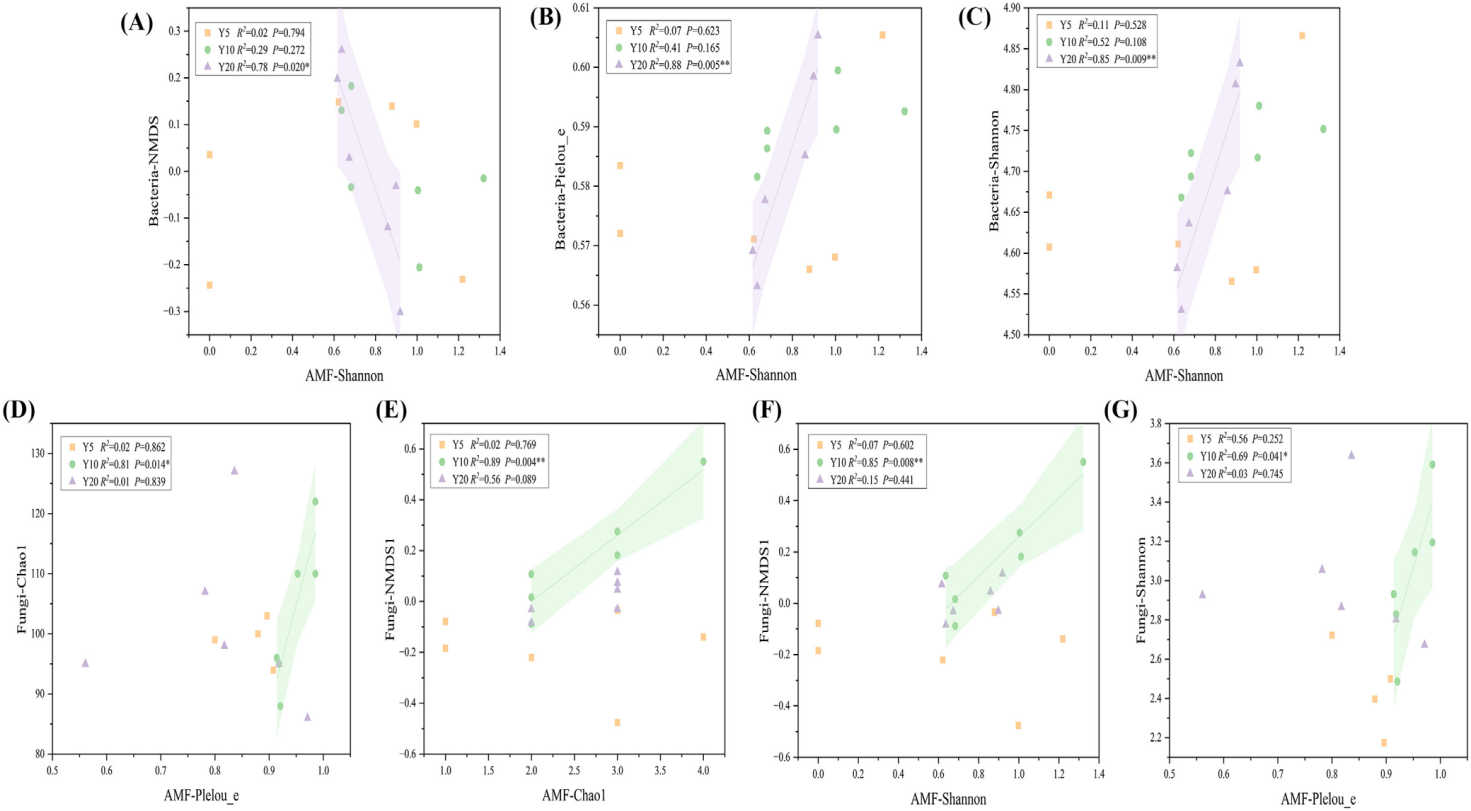
Relationships between AMF diversity and soil microorganism diversity (Y5: Planting 5 years; Y10: Planting 10 years; Y20: Planting 20 years). (A–C) the relationships between AMF and bacteria; (D–G) the relationships between AMF and fungi. Solid lines are statistically significant, ** indicates significant differences at *p* < 0.01 level, * indicates significant differences at *p* < 0.05 level. The shaded areas show the 95% confidence interval of the fit.

The correlations between AMF diversity and other soil microbes varied across the chronosequence. In the planting‐Y20, bacterial community composition (NMDS; *R* ^2^ = 0.85, *p* = 0.009) exhibited a negative correlation with the AMF Shannon index (Figure 7A), whereas bacterial Shannon (*R* ^2^ = 0.78, *p* = 0.020) and Pielou_e indices (*R* ^2^ = 0.88, *p* = 0.005) were positively correlated with it (Figure 7B,C). In the planting‐Y10, fungal Chao1 (*R* ^2^ = 0.81, *p* = 0.014) and Shannon indices (*R* ^2^ = 0.89, *p* = 0.004) were positively correlated with the AMF Pielou_e index (Figure 7D,G). Fungal community composition exhibited a positive correlation with the AMF Chao1 (NMDS; *R* ^2^ = 0.85, *p* = 0.008) and Shannon indices (*R* ^2^ = 0.69, *p* = 0.041) (Figure 7E,F).

### 3.6 Impact of Edaphic Factors Dynamics to AMF Community Assembly

**FIGURE 8:**
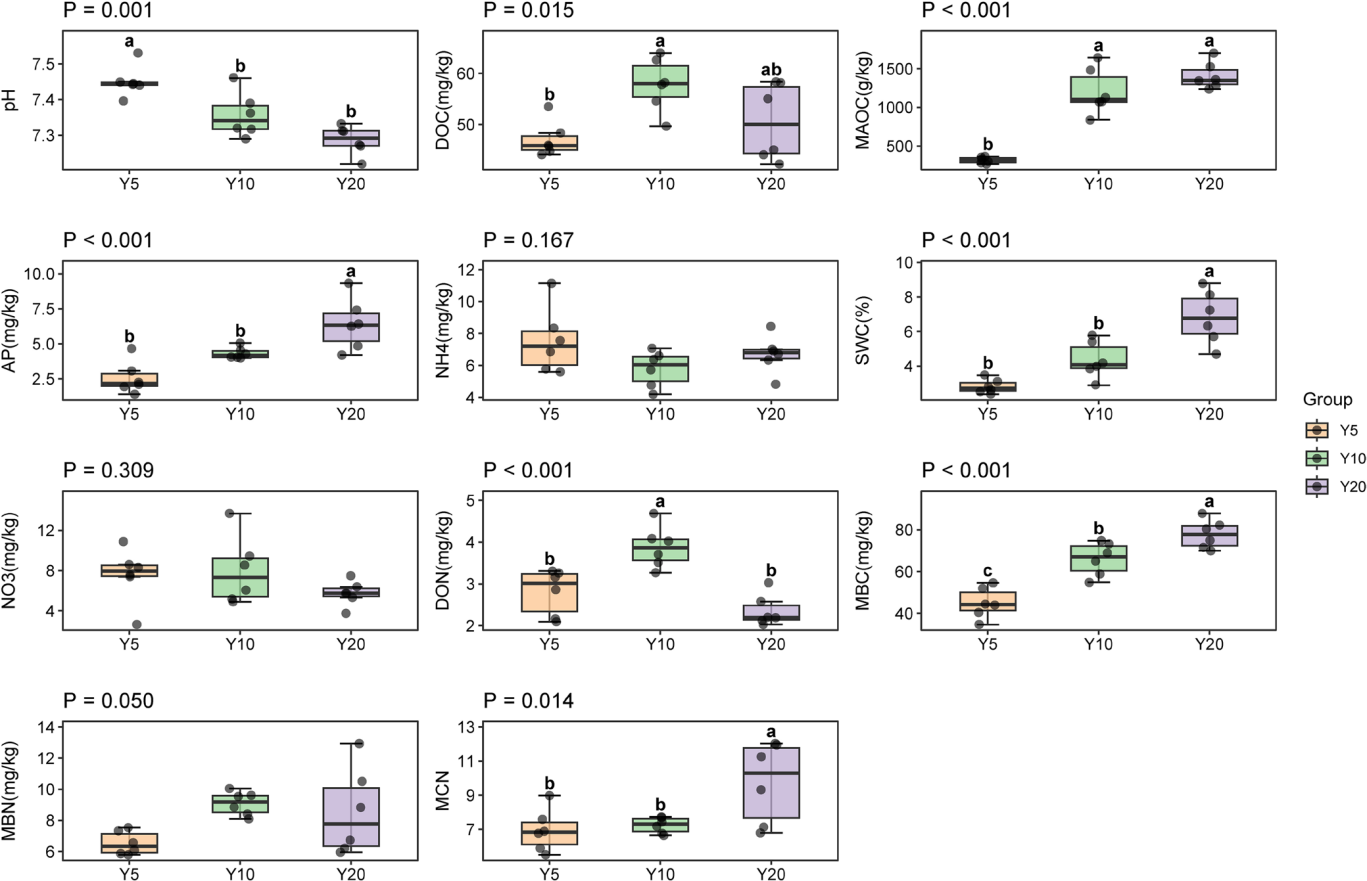
Variations in soil physicochemical properties along with restoration chronosequence (Y5: Planting 5 years; Y10: Planting 10 years; Y20: Planting 20 years). Box plots illustrate the distribution of 11 soil edaphic factors and individual dots represent raw data points (*n* = 6). The *p*‐value displayed at the top of each panel indicates the significance level from a One‐way ANOVA; “NS” denotes no significant difference (*p* > 0.05). Different lowercase letters (a, b, c) above the boxes indicate significant differences between groups (*p* < 0.05) based on Tukey's test; groups sharing the same letter are not significantly different. AP, available phosphorus; DOC, dissolved organic carbon; DON, dissolved organic nitrogen; MAOC, mineral‐associated organic carbon; MBC, microbial biomass carbon; MBN, microbial biomass nitrogen; MCN, microbial biomass C/N ratioNH4, ammonium nitrogen; NO_3_, nitrate nitrogen; PH, Soil acidity/alkalinity; SWC, soil water content.

**FIGURE 9:**
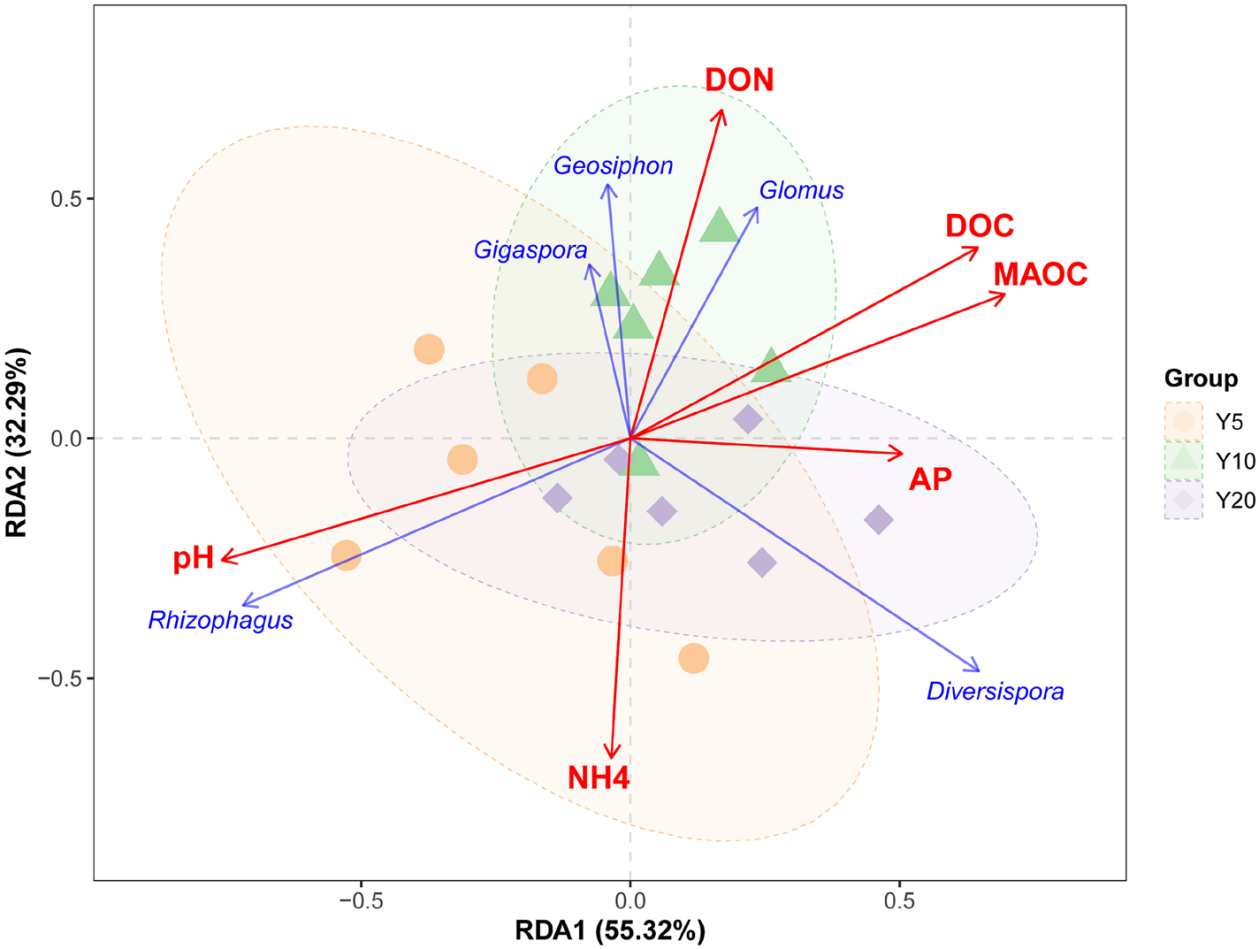
Redundancy analysis (RDA) illustrating the relationships between AMF community structure and soil physicochemical properties (Y5: Planting 5 years; Y10: Planting 10 years; Y20: Planting 20 years). Symbols of different colors and shapes represent samples from different treatments, dashed ellipses indicate the 95% confidence intervals for each group. Red arrows represent the explanatory variables. Blue arrows represent the response variables. The length of the arrows indicates the degree of correlation with the community structure, and the angle between arrows reflects the correlation type (acute angles indicate positive correlations, while obtuse angles indicate negative correlations). The RDA1 and RDA2 axes account for 55.32% and 32.29% of the total variation, respectively. AP, available phosphorus; DOC, dissolved organic carbon; DON, dissolved organic nitrogen; MAOC, mineral‐associated organic carbon; NH4, ammonium nitrogen; pH, soil acidity/alkalinity.

Edaphic factors varied significantly along the restoration chronosequence. Apart from a decrease in pH (*p* < 0.001), MAOC, AP, and SWC accumulated (*p* < 0.001), and the content of DOC (*p* < 0.05) and DON (*p* < 0.001) peaked in the planting‐Y10 (Figure 8). Redundancy analysis explained 87.61% of the total variation. *Rhizophagus* exhibited a positive correlation with soil pH in the planting‐Y5; *Glomus*, *Gigaspora*, and *Geosiphon* were mainly driven by DON and DOC in the planting‐Y10, and *Diversispora* was positively correlated with AP in the planting‐Y20 (Figure 9).

## 4 Discussion

### 4.1 Soil Bacterial and Fungal Communities Assembly Mechanisms and Sensitivity

Our findings revealed a striking divergence in the successional trajectories of rhizosphere microbes that fungal communities exhibited pronounced structural shifts and sensitivity to the restoration chronosequence, whereas bacterial communities remained relatively stable in structure despite significant changes in edaphic factors. We attribute this divergence to the fundamental differences in their assembly mechanisms, metabolic plasticity, and dependence on host plant resources.

Contrary to the expectation that bacteria are strongly shaped by edaphic factors (Guo et al. 2019; Sun 2020; Yang et al. 2023), bacterial community structure showed minimal variation across the chronosequence. This stability can be firstly explained by their broad metabolic plasticity in structure, diversity, and biomass (Uroz et al. 2016; Wang, Zhang, et al. 2023). Secondly, as demonstrated by the assembly process, the deterministic assembly of bacterial community initially stemmed from strong selective pressures imposed by nutrient scarcity in soil and limited plant‐derived resources, favoring taxa with broad physiological tolerances to colonize oligotrophic soils firstly (Zhang et al. 2018). However, the “resource island” effect created by *S. cupularis* establishment during desertified alpine meadows restoration enhanced the spatial heterogeneity of soil resources both horizontally and vertically (Li, Shen, et al. 2021). The accumulated nutrients and moisture as we have observed appear to alleviate environmental selection pressure over time, facilitating a shift towards stochastic assembly later. This trajectory diverges from observations in Wu et al.'s (2023) graminoid‐dominated systems where intense nutrient competition maintained deterministic dominance consistently based on a short‐term (4‐year) study of *Elymus nutans* or *Avena sativa* .

In contrast, fungal communities exhibited significant shifts in diversity and structure, mirroring the rapid successional trajectory of the host plant. As shrub biomass and canopy cover expanded along the *S. cupularis* chronosequence (Hu et al. 2018), the increasing shrub age led to substantial accumulation of shoot and root biomass, thereby increasing litter input (McClaran et al. 2008; Li, Shen, et al. 2021; Shu 2022). Fungal sensitivity was predominantly structured by plant community characteristics in most studies (Guo et al. 2019; Sun 2020; Yang et al. 2023) via specific symbiotic and parasitic linkages (Zhang, Cao, et al. 2021; Wang, Zhang, et al. 2023). The plant‐derived carbon fractions likely favored opportunistic taxa such as *Rhizopus,* which is adapted to labile substrates from root exudates as a typical r‐strategist and tends to be enriched in rhizosphere soil because its aseptate, easily detached hyphae adhere to root surfaces and disperse with soil particles (Deacon 2013). These contributed to explain the dominance of Mucoromycota caused by *Rhizopus* abundance. As recalcitrant nutrients accumulated in later stages, succession shifted towards complex organic matter decomposers, specifically Basidiomycota with superior enzymatic capabilities for degrading lignin and cellulose (Li, Yang, et al. 2021; Zhang, Liu, et al. 2021; Yang et al. 2023). Furthermore, SWC emerged as an environmental filter, influencing the dispersal and germination of fungal propagules (Wan et al. 2024; Zhang et al. 2024), thereby potentially driving the observed community variability. Additionally, fungal community assembly was statistically dominated by stochastic processes throughout the chronosequence, and this stochasticity was largely attributable to dispersal limitation. Unlike bacteria, whose high motility, small cell sizes, and even broad niche width facilitate homogenization across heterogeneous environments, fungal dispersal is physically constrained by spatial heterogeneity involving hyphal growth forms and larger propagule sizes (Gong et al. 2023; Wu et al. 2023), particularly in wind‐eroded alpine settings (Nemergut et al. 2013). This dispersal limitation creates high β‐diversity as observed in our results.

Meanwhile, the stochasticity ratio in microbial community assembly significantly positively correlates with network stability (Zhang and Adamczyk 2025). The high modular structure (modularity coefficients > 0.4) and prevalence of positive interactions (> 90%) likely provided diverse ecological niches that mitigated competitive exclusion for space and resources (Li et al. 2019), suggesting that shrub establishment could stabilize the microbial community by alleviating environmental stress. Furthermore, the higher complexity of bacterial networks compared to fungal ones supports the notion that complex network topologies offer greater resistance to environmental disturbances (Santolini and Barabási 2018). Given the absolute dominance of bacteria among soil microbes with a proportion exceeding 95% (Dao et al. 2023). We suggest that the robust stability of the bacterial network provides a distinct line of evidence for the ecological recovery of desertified land.

### 4.2 Mechanisms of AMF Community Assembly and the Functional Transition of Dual‐Mycorrhizal Strategies

As an important and special type of microbial functional group, the AMF community exhibited temporal dynamics characterized by a “hump‐shaped” diversity trajectory peaking at the 10th year after planting, align with global patterns of microbial recovery in degraded ecosystems (Zhang, Cao, et al. 2021). This peak likely represents an optimal restoration window during which resource availability supports maximal niche differentiation before competitive exclusion intensifies.

The rapid growth of *S. cupularis* provided abundant available nutrients, as evidenced by a pronounced pulse of DOC and DON in the planting‐Y10. While AMF cannot directly utilize DON, its presence may signal a phase of active nitrogen cycling and a permissive plant carbon allocation pattern, which likely fueled the proliferation of *Glomus* and the carbon‐demanding K‐strategist *Gigaspora,* which invests heavily in highly branched extraradical hyphae (Smith and Read 2008), as the succession of AMF is directly driven by host ontogeny (Šmilauer et al. 2021; Nash et al. 2025). We propose that the extensive hyphal architecture of *Gigaspora* may function as a physical scaffold (“hyphal highways”; See et al. 2022), thereby facilitating the co‐occurrence and potential interactions of diverse soil fungi even without numerical dominance, which explains the strong AMF‐fungal correlation and network complexity observed in the planting‐Y10. However, ecosystem maturation led to a fundamental shift in belowground resource economics. The accumulation of organic carbon suggests a transition to an “organic nutrient economy” (Phillips et al. 2013), which created a state of relative N limitation for the host plant as indicated by a significant increase in MAOC and concurrent, significant decline in DON. Under these conditions, maintaining high‐biomass AMF like *Gigaspora* yields diminishing returns, leading to its competitive exclusion.

In fact, this resource trade‐off extends beyond intraspecific AMF dynamics to interspecific mycorrhizal relationships. Our results confirmed that *S. cupularis* supported dual mycorrhizal associations, implying that the key factor in the evolution lies in the dynamic plasticity of host‐mycorrhizal associations (Rog et al. 2025). The bioavailable inorganic nutrients required by AMF became stagnant with restoration. As our study also found, after 10 years of *S.cupularis* cultivation, most bioavailable nutrients declined, including NO_3_^−^‐N and DON, while low‐molecular‐weight DON can also be utilized by AMF under oligonitrophic conditions of the plateau. Consequently, the AMF pathway became energetically inefficient because AMF lack the enzymatic capacity to access these sequestered organic pools. At the same time, the host plant strategically reallocated carbon to Ectomycorrhizal (ECM) fungi, which possess superior capabilities to produce oxidative enzymes to “mine” N directly from soil organic matter (Averill et al. 2014; Zhang and Adamczyk 2025) and exhibit greater affinity (Smith and Read 2008). This functional substitution leaves only stress‐tolerant taxa, *Glomus* and *Rhizophagus,* to persist in the root cortex based on “Symbiosis Cost” (Herre et al. 1999). This transition also elucidates the stage‐specific shift in microbial correlations. Surviving AMF appear to shift strategies in response to reduced carbon allocation. The positive correlation between AMF and bacterial diversity in this N‐limited, ECM‐dominated transitional environment suggests a “stress‐induced recruitment” of N‐fixing or mineralizing bacteria (e.g., *Bradyrhizobium*, identified in our network), potentially compensating for their limited to mine organic N (Zhang, Cao, et al. 2021; Duan et al. 2024). In return, AMF hyphae release exudates (sugars, lipids) to sustain bacterial metabolic activity (Lu et al. 2023). These interactions might be mediated either by the plant (i.e., AMF‐plant‐bacteria system) or soil nutrients (i.e., AMF‐soil‐bacteria system) (Yang et al. 2023; Sun et al. 2024).

Furthermore, the dominance of ECM fungi may induce the Gadgil effect (Averill et al. 2014), suppressing free‐living decomposers to conserve organic matter, which aligns with the significant accumulation of MAOC observed in our study. Thus, the microbial succession observed here is not a passive response to soil maturation, but an active, host‐mediated optimization of symbiotic trade‐offs. Although sufficient AP at the 20th year was detected, we predict that P may still become the main limiting factor as restoration continues, reaching the level observed in the 34‐year stands of Hu et al.'s (2018) that supports the P‐depletion hypothesis. Therefore, further temporal scale verification is needed.

Overall, our results highlight the time‐dependent regulatory role of AMF. While ECM may dominate the stable state, rapid AMF recovery plays a preeminent functional role in the critical early establishment phase. Consequently, we propose that restoration strategies should not merely focus on the climax state but must prioritize the targeted introduction of locally adapted, high‐efficiency native AMF strains (*Glomus* and *Rhizophagus* spp.) during the establishment phase to accelerate the construction of underground microbial networks and catalyze the rehabilitation of desertified alpine meadows.

## 5 Conclusions

Pioneer shrub enhanced the complexity of both soil bacterial and fungal networks, with fungal communities exhibiting greater sensitivity reflecting larger plasticity throughout the chronosequence. We revealed the time‐dependent regulatory role of AMF, acting as the primary orchestrator of rhizosphere microbial community assembly during the early phase‐peaking in the 10th year after planting, prior to the ecosystem's transition to a stable, ECM‐dominated state coupled with shifts in soil resource availability (e.g., rising MAOC and AP, as well as declining DON). However, the limited temporal scale in this study restricted a complete understanding of the long‐term response of dual mycorrhizal associations to restoration. Thus, future efforts should prioritize the identification of core functional strains, coupled with synergistic co‐inoculation of indigenous probiotic microbiota to enhance belowground interactions to accelerate the restoration of desertified alpine ecosystems.

## Author Contributions

**Xueqi Cai:** formal analysis (lead), visualization (lead), writing – original draft (lead). **Xingpeng Hu:** conceptualization (equal), investigation (equal). **Fei Yan:** validation (lead), writing – review and editing (equal). **Dongming Chen:** conceptualization (equal), writing – review and editing (equal). **Bingxue Xiao:** supervision (lead). **Xin Zheng:** validation (supporting). **Kangqi Zhang:** supervision (supporting). **Jiqiong Zhou:** methodology (equal). **Zhouwen Ma:** project administration (equal). **Feida Sun:** supervision (supporting). **Yan Peng:** validation (supporting). **Xiao Ma:** project administration (equal). **Jeyakumar Paramsothy:** writing – review and editing (supporting). **Ran Xue:** validation (supporting). **Lin Liu:** conceptualization (lead), data curation (supporting), writing – review and editing (lead).

## Funding

This work was supported by the National Natural Science Foundation of China (32571899), the Department of Science and Technology of Sichuan Province (2024NSFSC1965), the China Scholarship Council (202306910027), the Sichuan Forage Innovation Team Program (SCCXTD‐2026‐16), and Undergraduates' Innovative Entrepreneurial Training Plan of Sichuan Province (S202310626070).

## Conflicts of Interest

The authors declare no conflicts of interest.

## Data Availability

All data generated or analyzed during this study are made available at zenodo (DOI: 10.5281/zenodo.18258440).
